# Impact of Maternal High Stocking Density during the Dry Period on Dairy Calf Health, Behaviour, and Welfare

**DOI:** 10.3390/ani10060922

**Published:** 2020-05-26

**Authors:** Mayumi Fujiwara, Marie J. Haskell, Alastair I. Macrae, Kenneth M. D. Rutherford

**Affiliations:** 1 Global Academy of Agriculture and Food Security, University of Edinburgh, Midlothian EH25 9RG, UK; 2 Animal Behaviour & Welfare Team, Animal and Veterinary Science Research Group, SRUC, Edinburgh EH9 3JG, UK; Marie.Haskell@sruc.ac.uk (M.J.H.); Kenny.rutherford@sruc.ac.uk (K.M.D.R.); 3 Dairy Herd Health and Productivity Service, Royal (Dick) School of Veterinary Studies and the Roslin Institute, University of Edinburgh, Midlothian EH25 9RG, UK; A.I.Macrae@ed.ac.uk

**Keywords:** prenatal stress, stocking density, pre-weaning period, dairy calf behaviour

## Abstract

**Simple Summary:**

Negative impacts of stressful maternal experience during pregnancy on offspring health and behaviour have been reported in various mammalian species including humans, laboratory animals, and farm animals. This study investigated the effect of limited space allowance for dairy cows during late gestation on the growth and behaviour of their offspring during the pre-weaning period. Our results indicated associations between maternal high stocking density and a higher frequency of social behaviours and increased behavioural reactivity to weaning in offspring. Maternal high stocking density also reduced behavioural reactions of healthy offspring to a painful procedure. However, there was no association between maternal high stocking density and offspring growth or behaviour in the first week of life. To our knowledge, this study is the first to attempt to demonstrate associations between maternal stocking density during late pregnancy and offspring behaviour in dairy cattle.

**Abstract:**

This study aimed to investigate the effect of maternal stocking density during late pregnancy (approximately 60 ± 4 days before calving) on offspring performance during the pre-weaning period. Forty-five dairy calves were born to cows that went through either industry minimum standards (H: *n* = 24, high stocking density) or more extensive space allowances (L: *n* = 21, low stocking density) during the dry period. Body weight and average daily gain during the pre-weaning period (day 1–49) were measured. Observations were made of: (i) activity levels (day 2–6); ii) the level of training required to use an automatic feeder, and behavioural reactions to the group environment (d7); (iii) feeding and social behaviour in the group pen (day 7–21); and (iv) responses to weaning (day 40–49) and disbudding (day 28+). Compared to L calves, H calves made more frequent social contacts with pen mates in the group pen (*p* = 0.003) and decreased their lying time around weaning (*p* = 0.045). Among the healthy calves, L calves displayed more severe behavioural reactions to the disbudding procedure (*p* < 0.001), a significant increase in salivary cortisol concentrations (*p* = 0.013), and more frequent pain-related behaviour (*p* = 0.036). This study indicated associations between maternal stocking density during late pregnancy and some welfare-relevant offspring outcomes during the pre-weaning period; these effects were found to be modulated by offspring health status.

## 1. Introduction

A number of studies have indicated that maternal experiences such as stress or suboptimal nutrition during pregnancy can affect foetal development [1,2]—for instance, via epigenetic changes [3]. Maternal stress, in particular, is known to affect offspring brain development [4]. The regions of the brain susceptible to maternal stress are associated with cognitive ability, emotions (fear and anxiety) and responses to stress [5]. Potential consequences of altered brain development include altered stress responsiveness and/or impaired learning and stress-coping abilities in aversive conditions [2,6]. Such effects may have important consequences for animal welfare in livestock production [7]. 

Studies on farm animal species also indicate that there are associations between maternal stress during pregnancy (prenatal stress) and offspring performance, behaviour and welfare (cattle [8]; pig [9]; sheep [10]; poultry [11]). Stressful treatments imposed on pregnant mothers (e.g., social mixing and isolation) have been shown to affect offspring’s behavioural and physiological responses to stress in sheep and pigs [12,13,14]. In cattle, maternal heat stress during late pregnancy had significantly reduced birth weight [15,16,17,18,19,20,21], serum IgG levels, and weaning weight [20] of dairy calves. Lay and colleagues reported that repeated transportation of cows throughout pregnancy altered the offspring’s physiological response to stress [22]. More recent studies also reported that repeated transportation of pregnant cows resulted in higher basal cortisol levels and more temperamental traits in offspring [23]. The findings from these studies would suggest that it is possible that certain maternal factors can affect foetal development in cattle. 

Pregnant dairy cows can experience various stressors as a part of routine management practices. During the dry period in particular, cows have the potential to experience increased stress due to changes in management within this period [24], as well as physiological, metabolic and hormonal changes as they approach parturition. Prepartum stress and stressful management practices during the dry period have been shown to negatively affect cow behaviour and subsequent performance (e.g., heat stress [25]; dry-off procedure [26]; overstocking [27]; frequent regrouping [28]). However, none of these studies investigated consequences of maternal stress or maternal stressful experiences due to dry cow management on offspring behaviour and welfare. Only studies on maternal heat stress reported the impact on offspring such as impaired calf growth and immune functioning [25]. More recently, Black and colleagues reported that maternal exercise during late pregnancy potentially increased offspring weaning stress, which may be mediated by increased levels of circulating cortisol in cows due to excessive exercise [29]. 

Dairy calves on commercial farms are normally artificially reared by humans. This unnatural environment requires calves to adapt to challenges associated with management practices, such as early separation from the dam and restricted milk feeding to enhance early starter intake. It would be advantageous if such calves were equipped with a greater ability to adapt to their environment, with stress-coping abilities that are not impaired by prenatal factors. 

The aim of this study was to investigate the effect of maternal high stocking density during late pregnancy on dairy calf growth, health, behaviour and welfare during the pre-weaning period. High stocking density is a common feature of large commercial dairy farms and has been shown to increase agonistic interactions among cows and disrupt feeding behaviour [30,31]. Calves in the current study were born to cows that experienced either a high or low stocking density at the feed face and the lying area throughout the dry period [32]. The high stocking density treatment resulted in more competition at the feed face and a reduction in feeding duration during the peak feeding period compared to the low stocking density group. Cows in the high stocking density group also took longer to start feeding when freshly delivered feed was put out and spent more time standing in the alley during the peak feeding period (three hours after feed delivery) [32]. 

Although there were no differences between treatment groups in maternal metabolic profiles or faecal glucocorticoid metabolites, cow behavioural differences between the two treatment groups suggested that they were sufficient to alter cow biology. Therefore, the current study hypothesised that alterations to maternal stocking density in late pregnancy would negatively affect offspring performance under commercially relevant situations. Aspects of progeny performance investigated included calf body weight, health and growth in the pre-weaning period, the activity level of calves in early life, adaptability of calves to an automated-feeding and group-housing system, reactions to handling and weaning, and behavioural and physiological responses to disbudding.

## 2. Materials and Methods

All experimental procedures were approved by the SRUC Animal Welfare and Ethical Review Body (Animal Experiment Number: AE 41-2014). Calves used in the experiment were born between 15th January and 23rd August 2015 from cows used in a stocking density experiment at the Crichton Royal Farm (Dumfries, UK) in Scotland’s Rural College (SRUC). Forty-eight Holstein cows were dried off 8–9 weeks before their expected calving date and allocated to either high (H) or low (L) stocking density groups from then until calving (H: 1 cow had 0.5 headlock or 0.3 m feed-face space + 1 cubicle or 6 m^2^ lying area; L: 1 cow had 1 headlock or 0.6 m feed-face space + at least 1.5 cubicle or 12 m^2^ lying area). Full details of maternal treatment and outcomes are described in [32]. 

### 2.1. Animals, Housing and Feeding

Forty-five calves were studied from birth to weaning (H group: *n* = 24, L group: *n* = 21). The H group comprised 11 beef cross calves (bull = 5, heifer = 6) and 13 dairy calves (bull = 6, heifer = 7), and the L group comprised 10 beef cross calves (bull = 6, heifer = 4) and 11 dairy calves (bull = 5, heifer = 6). As there were no existing data suitable for a power calculation, sample size was determined from similar studies in the literature [19,20,21,22,33,34]. All calves were managed in the same way irrespective of maternal treatment. Calves were born in a straw-bedded yard, fed four litres of pooled colostrum via an oesophageal tube within four hours after birth, and stayed with their dams for two to eight hours. Calving was supervised by experienced farm staff, and assistance was provided where necessary by either farm staff or a veterinarian. Calves were then weighed (BW birth) and moved to a straw-bedded individual hutch (1.0 m × 1.4 m × 1.2 m) with a straw-bedded front yard (1.2 m × 1.4 m) in a calf barn. Calves were fed three litres of milk twice a day (8:00 and 15:30) from a bucket with a rubber teat. Non-pasteurised whole milk was provided until the end of February 2015, and milk replacer (VITAMILK Omega Gold: 23.0% Protein, 18.0%, Oil 18.0%, ForFarmers UK Limited, Bury St Edmunds, UK) was fed from the beginning of March 2015 due to a change in calf feeding management on the farm. Water and calf starter pellets (VITA Start: 18.0% Crude Protein, 11.5% Crude Fibre, Crude Fat 4.0%, 1.0% Ca, 0.5% P, 0.3% Na, 0.3% Mg, Vitamin E 60 IU/kg, ForFarmers UK Limited) were provided ad libitum from buckets on the front wall of the yard. 

The health condition of calves was monitored every day by farm staff, and any incidences of disease and associated treatments were recorded. The most common diseases that pre-weaned calves were treated for were respiratory disease and diarrhoea. To prevent *Cryptosporidium parvum* infection, Halofuginone (Halocur^®^; MSD Animal Health, Milton Keynes, UK) was given after morning feeding from d1 to d6 according to the manufacturer’s recommendations. No vaccination was given at this stage.

At d7.3 ± 0.4 standard deviation (SD), calves were weighed (BW d7) and moved to a straw-bedded group pen (6.6 m × 5.0 m) with access to an igloo shed (4.5 m × 5.0 m × 1.7 m) at the end of the pen. Calves were moved before the morning feeding (between 7:30 and 10:30) and were then trained to feed from an automatic milk feeder (H&L 100, Holm & Laue GmbH & Co. KG, Westerrönfeld, Germany). Milk replacer was fed from the automatic milk feeder that was attached to the front wall of the group pen, and a maximum of 6.0 L was provided per day. The maximum milk allowance was set at 1.0 L for each feeder visit to distribute meals throughout the day, and calves that drank 1.0 L at a single visit were not dispensed milk for the following 100 min. Calf starter pellets and straw were provided ad libitum from troughs attached to the pen wall, and water was available from an automatic water dispenser. Group structure was dynamic with calves entering and leaving depending on the date of introduction from the hutches and subsequent weaning dates. Group size ranged between two and 15 calves per group, except for the period from 2nd to 18th of March 2015 when four calves were put into a double-sized group pen with one milk feeder, two straw and starter troughs and two automatic water dispensers (two group pens were combined). This was due to a shortage of milk feeders, and the maximum group size of this pen was 25 calves. 

Calves over 28 days old were disbudded with an electric dehorner by a veterinarian. Cornual nerves were anaesthetised 10 min prior to disbudding with 3.0 mL of 50 mg/mL procaine hydrochloride (Adrenacaine, Norbrook^®^ Laboratories (BG) Ltd., Northamptonshire, UK), and no separate analgesics were administered. Weaning was completed at 49 days of age with a gradual reduction in daily milk allowance from 6.0 to 0.0 L over 10 days. After weaning, calves were weighed (BW wean) and removed from the group. 

### 2.2. Data Collection and Data Processing

#### 2.2.1. Body Weight and Average Daily Gains

The health condition of calves was monitored every day by farm staff, and any occurrence of disease and associated treatments were recorded. Average daily gains (g/day) in the hutch (day 1–6: ADG hutch), in the group pen (day 7–49: ADG group), and in the pre-weaning period (day 1–49: ADG pre-weaning) were calculated by dividing BW gains during the corresponding periods by days in the corresponding periods. On d7, blood was collected from the jugular vein into a 10 mL sterile plain tube and centrifuged at 3000× *g* for 10 min, and sera were stored at −20 °C until analysis. Concentrations of serum immunoglobulin G (IgG) were measured using a commercial kit (Bovine IgG ELISA kit; Biopanda Reagents, Belfast, UK) according to the manufacturer’s instructions.

#### 2.2.2. Activity Levels

Activity data loggers (AX3, Axivity, Newcastle Upon Tyne, UK) were used to measure the activity of calves. The logger was attached to one of the hind legs of each calf from day 1 to 9 of life and was set to record position (lying, standing, and intermediate) twelve times per second. On day 9, the data were downloaded and then converted to represent durations (seconds) of each position using SQL Server Management Studio (Microsoft). Daily lying proportion (LP) from day 2 to 6 in the hutch (LP hutch) and LP in the group pen (LP group) on group day 1 (0–24 h in the group pen) and group day 2 (24–48 h in the group pen) were calculated. The data for d1 were often incomplete (less than 24 h) and therefore not included in the analysis.

#### 2.2.3. Training for the Use of an Automatic Milk Feeder

On the morning when calves were moved to a group pen, they were immediately trained to drink from the milk feeder. A training protocol was created to ensure that all calves were taught in the same manner. Calves were trained again at the afternoon feeding time (15:30) unless they had already independently drank milk (self-fed) before then. The training was continued on subsequent days in the morning (08:00) and in the afternoon (15:30) until the calf self-fed. The number of trainings required for calves to self-feed was recorded for each calf (training counts). 

#### 2.2.4. Reactions to a Novel Group Environment, Behaviour for the First Two Weeks in the Group Pen and Reactions to Weaning

Video cameras (Hi Res Bird Box Camera, 700TVL Sony EFFIO CCD, IR Night Vision, SpyCamera CCTV Ltd., Bristol, UK) connected to a Geovision system (version8, Geovision Inc., Taipei, Taiwan) were used to monitor calf behaviour in the group pen from 06:00 to 18:00 daily until all calves were weaned. All of the behavioural observations were performed by a single observer after a training period to ensure intra-observer agreement, in which video recordings of ten calves were watched three times until more than 90% agreement of the measures was achieved.

Reactions of calves to the novel environment with novel companions on first introduction to the group pen were assessed by continuous behavioural observation for 30 min, from the time when the whole body of the calf was out of the feeder (i.e., when the calf finished its first meal). Location in the pen, posture, exploratory and social behaviour of calves were recorded using Observer^®^ XT 12.5 (Noldus Information Technology b.v., Wageningen, The Netherlands), according to a pre-determined ethogram (Table 1). Latencies for each calf to first perform the behaviours walk, run, explore, enter the igloo shed, lie down, initiate social contact and receive social contact from a pen mate(s) were calculated. Time spent inside the igloo shed and in the straw yard, time spent standing inactive, and time spent engaging in the following behaviours (walk, run, explore and social contact) were also calculated. Frequencies of walking, running, exploring both/either in the igloo shed and/or the straw yard, and social contact (initiated or received) were also calculated. 

Five-minute scan sampling was conducted on individual calves on four different days (Gday 1–4) during the first two weeks in the group pen: Gday1 (either group day 1 or 2), Gday2 (any day between day 3 and 6 in the group pen), Gday3 (between day 8 and 10 in the group pen), and Gday4 (between day 11 and 14 in the group pen). Observations were conducted between 13:30 and 16:30, when the daytime behavioural pattern of calves was well represented with minimal human intervention. Calf location, posture, proximity to neighbouring calves for each calf and whether a calf’s muzzle or body was touching other calves (Table 1) were recorded for each scan. 

The behavioural reactions to weaning were video monitored for three hours (13:30–16:30) on three occasions: on day 40 or 41 (Wday 0: before weaning started), on day 45 or 46 (Wday 1: in the middle of the weaning process) and day 49 or 50 (Wday 2: when weaning was completed). Location of the calf, posture, and human intervention were recorded by five-minute scan sampling (Table 1). 

Data from the five-minute scan sampling for group pen behaviours (the first two weeks and around weaning) were converted to proportions of the total number of scans (i.e., the number of scans for the locations, postures, touching and proximity were divided by the total number of scans (*n* = 37)).

#### 2.2.5. Behavioural and Physiological Reactions to Disbudding

Calves (age: means ± SD = 36.9 ± 7.5 days; range = 28–56 days) were disbudded between 10:00 and 11:00. Each calf was put into a crush and its cornual nerves were blocked with local anaesthetic (LA), as previously described. Ten minutes later, the calf was disbudded with an electric dehorner.

Once the whole body of a calf was in the crush, reactions to LA administration (30 s) and reactions to the disbudding procedure (1.5 min) were observed. The frequencies of head movement [37] and kicking (a calf quickly lifting a hind hoof up and down, often making a noise when the hoof touched the wall or the floor of the crush) were recorded.

Frequencies of pain-related behaviour for individual calves were collected by 10 min live observations approximately 3.5 h (200 ± 20 SD min) after the procedure (when the effect of local anaesthesia started to wear off: [38]), and 6 h (364 ± 13 SD min) after the procedure (when the effect of local anaesthesia was predicted to disappear). Behaviours collected were head shaking, ear flicking, and head rubbing (for behaviour definitions, see [37] for head shaking; [38] and [39] for ear flicking and head rubbing). 

Salivary samples were collected from all calves using cotton swabs 24 h before the procedure (baseline), and 30 min, 4 h and 8 h after the disbudding. Cotton swabs were centrifuged at 3000× *g* for 15 min and extracted saliva was stored at −20 °C until analysis. Concentrations of salivary cortisol were measured using a commercial kit (Cortisol ELISA (Saliva), ALPCO^®^, Salem, NH, USA) as per the smanufacturer’s recommendations.

### 2.3. Statistical Analysis

All statistical analyses were performed using Genstat^®^ 16th Edition (VSN International Ltd., Hemel Hampstead, UK). Test statistics, *p* value (*p* ≤ 0.1), means or predicted means and standard errors of means (SEMs) are reported. For all statistical models, residual values were plotted to examine normality. A log transformation was used where necessary, and back-transformations of predicted means and corresponding 95% confidence intervals [95% CIs] are reported for transformed data. Due to a technical problem, data were missing for some calves (activity levels: *n* = 8, training record: *n* = 3, behaviour in the group pen: *n* = 10), and these calves were not included in the subsequent behavioural analyses.

#### 2.3.1. Individual Statistical Model Development

Individual statistical models were built for each of the data analyses to examine the effect of treatment, after being adjusted for characteristics of calves (gender, breed, maternal parity), disease incidence in the hutch/group pen (treated or not treated), season, and potential confounding effects for each of the outcomes. Potential confounding factors included training record (assessed for BW wean, ADG group, ADG pre-weaning), sampling timing (assessed for LP, group pen behaviour and salivary cortisol), age at introduction of a group pen (assessed for training count, reactions to a group environment), group size and human presence (assessed for group pen behaviour). Training record and group size were included as covariates. These variables were each firstly tested in a univariate analysis and were fitted in a multivariate model if they had *p*-values less than 0.25 [40]. Four calves that stayed in the double sized pen were included in the dataset, as the analyses with or without these calves did not change the outcome. Interactions between treatment and other variables of interest (*p* < 0.25) were fitted for all of the analyses by backward stepwise selection. When there were significant differences (*p* < 0.05) in a variable with more than two levels or significant interactions (*p* < 0.05), a post-hoc analysis (Fisher’s unprotected least significant difference test) was conducted to investigate the direction of the effect.

#### 2.3.2. BW, ADG, IgG, Training Count, LP, Reactions to a Novel Group Environment

A general linear model (GLM) was used to analyse BW birth, BW day 7, BW wean, ADG hutch, ADG group, ADG pre-weaning, and IgG levels. Proportional hazards (Cox) regression was used to analyse reactions to a novel group environment (latency), and the likelihood of the behaviour occurring within 30 min is reported as a hazard ratio (HR, H group as a reference level) with corresponding 95% CIs. The model for the latency of reactions to a novel group environment was adjusted for pen location.

A linear mixed model using restricted maximum likelihood (REML) procedures was used to analyse LP (hutch, group) and reactions to a novel group environment (duration and frequency). A generalised linear mixed model (GLMM) with a Poisson distribution and logarithm link function was used to analyse the training count data. The model for LP hutch included day in the hutch (day 2–6) as well as treatment and aforementioned factors as fixed effects and calf as a random effect. The final model for LP group included day in the group pen (group day 1, day 2), with random effects including calf and treatment nested within pen location (pen/treatment). Random effects for training count, duration and frequency of behaviour in a novel group environment were pen/treatment. The duration of social contact was analysed following a log transformation.

#### 2.3.3. Behaviour in the First Two Weeks in the Group Pen and around Weaning

REML was also used to analyse behaviour in the first two weeks (proportions of scans in which each calf was observed lying, standing, and being close to neighbouring calves in the first two weeks), and behaviour around weaning (the count of milk and starter feeder visits, proportions of scans in which a calf was observed lying). Proportions of the locations and touching showed skewed distributions and transformation of the raw data did not fulfil the assumption of normality. Therefore, GLMM was used with a binomial distribution (binomial total = 37) and a logit link function. The number of times calves were recorded at the water dispenser and the straw feeder were excluded from the analyses due to sparse observations.

The final models for behaviour in the first two weeks in the group pen and around weaning always included observation day (Gday 1–4 or Wday 0–2) as fixed effects, with calf and pen/treatment as random effects. In order to assess the changes in behaviours during the weaning process, interactions between observation day and factors of interest were independently tested, and significant interactions (*p* < 0.05 after being adjusted for other variables) were included in the final model for the behaviour around weaning as fixed effects.

#### 2.3.4. Behavioural and Physiological Reactions to Disbudding

The frequency of head moving and kicking were summed for each of the observations (during LA administration and the disbudding procedure) and were analysed using REML, including veterinarian ID as a random effect. Pain-related behaviours (head shaking, ear flicking and head rubbing) were summed for each of the observations (3.5 h and 6 h post-disbudding) and analysed using GLMM with a Poisson distribution using a logarithm link function. Veterinarian ID and pen/treatment were included as random effects. REML was used to analyse the change in concentrations of salivary cortisol 24 h pre-disbudding (baseline), and 0.5 h, 4 h and 8 h post-disbudding following a log transformation. The model included an interaction between treatment and sampling timing, as the main interest of this analysis was to assess any potential maternal treatment effect on changes in the salivary cortisol level before and after disbudding. Interactions between treatment, sampling timing and any potential confounding factors were tested independently, and significant interactions (*p* < 0.05) were left in the final model. Calf, veterinarian ID and pen/treatment were included as random effects.

## 3. Results

### 3.1. BW, ADG, Health and IgG Levels

There was no significant effect of maternal treatment on weight at any time point, ADG during the different phases, or serum IgG level at d7 (Table 2). Maternal treatment also did not affect the prevalence of respiratory disease or diarrhoea (H group: treated = 13, not treated = 11 calves, L group: treated = 13, not treated = 9 calves; *p* = 0.841). 

There was a significant effect of disease on BW and ADGs. Calves that were treated for illness in the pre-weaning period had a lower body weight at weaning compared to calves that required no treatment (treated: 66.1 ± 1.5 kg, not treated: 73.8 ± 1.8 kg, *Wald* = 9.5, *p* = 0.004). Calves that were treated in the group pen grew slower than calves that were not treated in the group pen (treated: 533.0 ± 31.2 g/day, not treated: 631.5 ± 31.2 g/day, *Wald* =4.4, *p* = 0.045). Disease incidence in the whole pre-weaning period did not affect pre-weaning growth, but there was a tendency for a slower growth in treated calves compared to non-treated calves (treated: 458.1 ± 26.7 g/day, not treated: 537.2 ± 31.8 g/day, *Wald* = 3.3, *p* = 0.079).

### 3.2. Activity Levels and Training Count for the Use of an Automatic Milk Feeder

Maternal treatment had no significant effect on the LP from day 2 to 6 in the hutch (Table 2). However, day in the hutch significantly affected LP hutch, with LP being the highest on day 2 and gradually decreasing until day 6 (Table 2). Calves that were treated for disease whilst in the hutch lay down for a significantly larger proportion of the day compared to calves that were not treated in the hutch (treated: 0.89 ± 0.02, not treated: 0.82 ± 0.001, *F_1,34_* = 10.6, *p* = 0.003). Maternal treatment did not affect LP in the group pen, but a significant interaction was found between treatment and day in the group pen (Table 2). L calves increased their lying proportion from group day 1 to group day 2 (*p* = 0.017), whilst the LP of H calves did not change from the group day 1 to group day 2. Calves that received veterinary treatment in the hutch had a greater LP in the group pen (0.83 ± 0.03) compared to healthy calves (0.78 ± 0.01, *F_1,31_* = 4.2, *p* = 0.048). Maternal treatment did not affect training counts (H: 2.2 ± 0.6 times, L: 2.5 ± 0.6 times, *F_1,35_* = 0.3, *p* = 0.624). 

### 3.3. Reactions to the Group Pen

There was no difference between treatment groups in the latencies of calves to perform any of the behaviours observed (Supplementary Table S1). However, L calves tended to start exploring the pen faster than H calves (HR = 2.13 [0.97−4.66], *p* = 0.061). There was no significant effect of treatment on the times spent engaging in any of the behaviours in the first 30 min after introduction to the group pen (Supplementary Table S2). There was a significant interaction between treatment and group size on the duration of walking (*F_1,30_* = 7.6, *p* = 0.010) and running (*F_1,27_* = 5.8, *p* = 0.023). Compared to H calves, L calves spent less time walking (−0.3 ± 0.1 min) and running (−0.1 ± 0.1 min) for each unit increase in group size. There was no significant difference between treatment groups in the frequency of walking, running, social and exploring behaviours in the 30 min after introduction to the group pen (Supplementary Table S2). However, L calves tended to receive social contact more frequently than the H calves (H: 12.8 ± 1.7, L: 17.1 ± 2.0 times, *F_1,29_* = 4.2, *p* = 0.051). 

### 3.4. Behaviour in the First Two Weeks in the Group Pen

**Figure 1:**
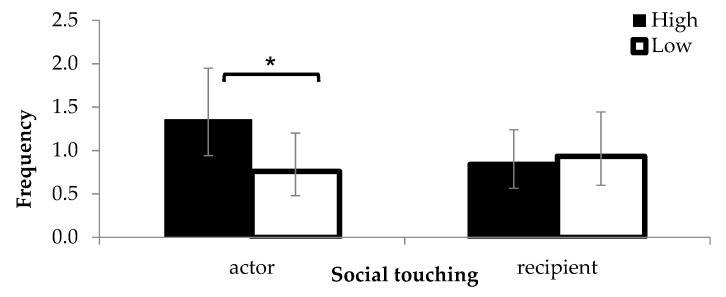
The number of social touching events observed as an actor or as a recipient (back- transformed means). Error bars indicate 95% Cis (confidence intervals). *: *p* < 0.05.

There was no statistically significant effect of maternal treatment on the proportion of time standing (H: 0.29 ± 0.02, L: 0.24 ± 0.03, *F_1,33_* = 0.4, *p* = 0.515), but the proportion of time lying tended to be lower in H calves compared to L calves (H: 0.67 ± 0.03, L: 0.74 ± 0.03, *F_1,6_* = 4.7, *p* = 0.075). H calves were more frequently observed touching other calves compared to L calves (*F_1,41_* = 9.7, *p* = 0.003, Figure 1). The number of social touching events as a recipient was not different between treatments (*F_1,113 =_* 0.3, *p* = 0.575, Figure 1), but a significant interaction was found between treatment and group size (*F_1,118_* = 5.6, *p* = 0.020). Compared to H calves, L calves were more likely to receive social touching from companions as group size increased (increase by 0.02 ± 0.03 probability per unit increase). Treatment did not affect the proportion of time spent close to a neighbouring calf (H: 0.3 ± 0.03, L: 0.30 ± 0.03, *F_1,118_* = 1.8, *p* = 0.187). 

### 3.5. Reactions to Weaning

**Figure 2:**
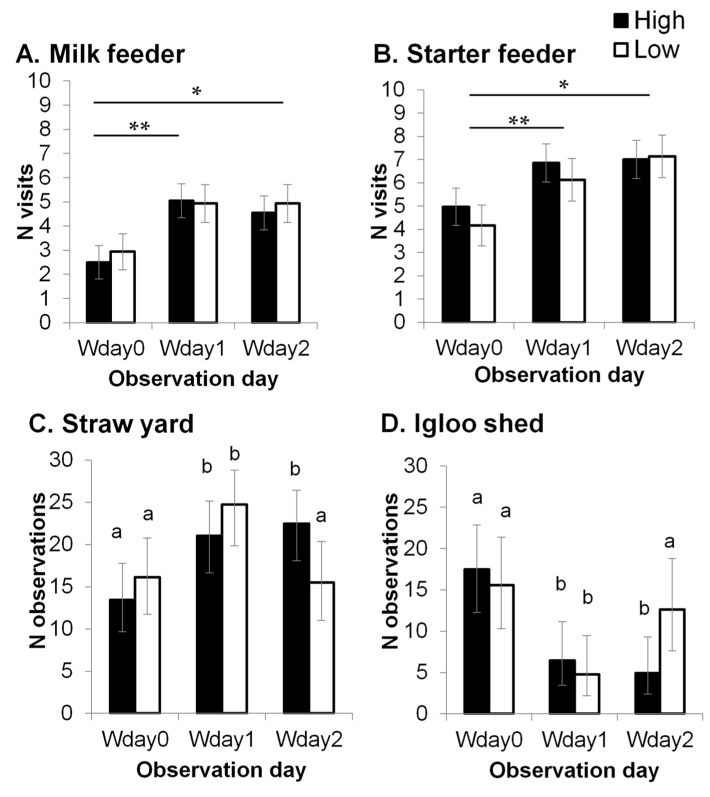
The number of milk feeder (**A**) and starter feeder (**B**) visits (predicted means ± SEM), and the number of times a calf was observed in the straw (**C**) and in the igloo shed (**D**) (back-transformed means and 95% Cis (confidence intervals).) before weaning started (Wday 0), in the middle of weaning (Wday 1) and when weaning was completed (Wday 3). *: significant difference between Wday 0 and Wday 2 (*p* < 0.01). **: significant difference between Wday 0 and Wday 1 (*p* < 0.001). ^a,b^: different letters indicate significant differences (*p* < 0.05).

There was no difference in the overall lying proportion between the H group (0.57 ± 0.02) and the L group (0.58 ± 0.03, *F_1,31_* = 0.1, *p* = 0.831), but a significant interaction was found between treatment and observation day (*F_2,72_* = 3.4, *p* = 0.039). The lying proportion for H calves was significantly lower in the middle of the weaning process (Wday 1: 0.49 ± 0.04), compared to the day before weaning started (Wday 0: 0.64 ± 0.04) and the day weaning was completed (Wday 2: 0.59 ± 0.04), but this difference was not observed in the L group (Wday 0: 0.58 ± 0.04, Wday1: 0.60 ± 0.04, Wday 2: 0.57 ± 0.04). This resulted in H calves having a significantly lower lying proportion than L calves on Wday 1 (*t* = 2.1, *p* = 0.045). The number of feeder visits was not affected by treatment, but the number of milk and starter feeder visits significantly increased from Wday 0 to Wday 1 and 2 (milk feeder: *F_2,71_* = 7.7, *p* < 0.001; starter feeder: *F_2,72_* = 6.6, *p* = 0.002, Figure 2A,B) in both the H and L groups.

No treatment effect was found on the number of times a calf was observed in the straw yard or in the igloo shed (*p* > 0.1). There was a significant effect of observation day in the number of times a calf was observed in the straw yard (Wday 0: 14.8 [11.8, 17.9], Wday1: 22.9 [19.6–25.9], Wday 2: 19.0 [15.7–22.3], *F_2,71_* = 6.4, *p* = 0.003), and in the igloo shed (Wday 0: 16.5 [12.6–20.5], Wday 1: 5.5 [3.4–8.7], Wday 2: 8.1 [5.3–11.9], *F_2,71_* = 14.0, *p* < 0.001). Moreover, a significant interaction between treatment and observation day was found in the number of times a calf was observed in the straw yard (*F_2,71_* = 3.6, *p* = 0.033), and in the igloo shed (*F_2,71_* = 3.8, *p* = 0.028). Calves were more frequently observed in the straw yard on Wday 1 compared to Wday 0 in both H (*p* = 0.015) and L (*p* = 0.012) groups (Figure 2C), and the opposite pattern was found in the igloo shed (H: *p* = 0.001, L: *p* = 0.002, Figure 2D). However, when weaning was completed, the number of times L calves were observed in the igloo shed and in the straw yard returned to the same level as the day before weaning started, whilst H calves were still more likely to be observed in the straw than in the igloo shed. This resulted in a significant difference between the treatments groups on Wday 2 in the number of times calves were observed in the igloo shed (*p* = 0.029) and the straw yard (*p* = 0.044).

### 3.6. Reactions to Disbudding

**Figure 3:**
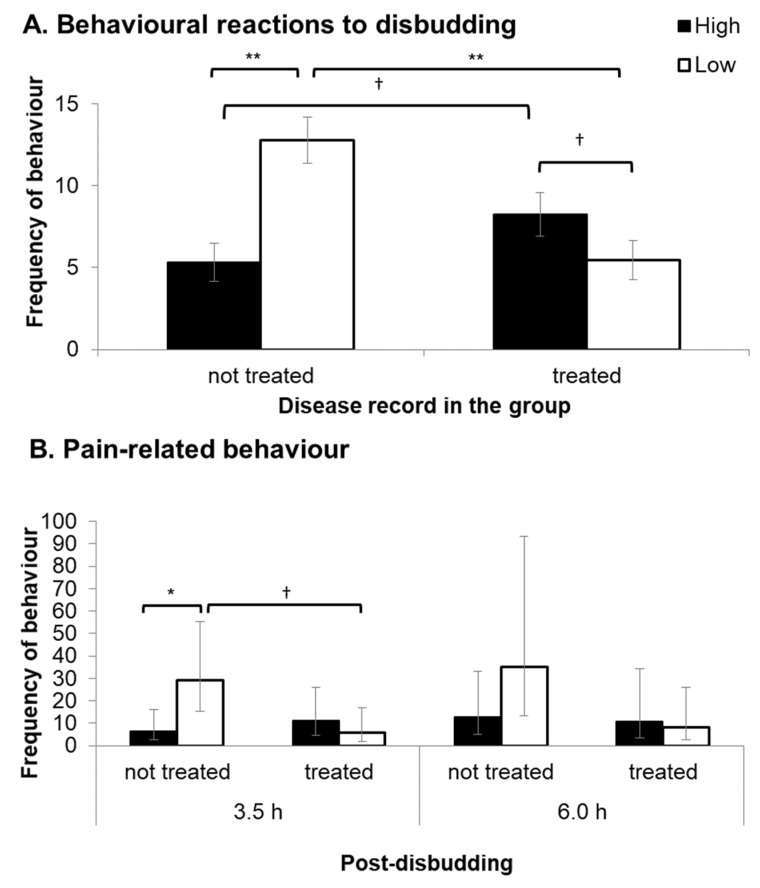
(**A**) Behavioural reactions (mean frequency ± SEM) to the disbudding procedure (head movement and kicking) for calves with no previous disease record in the group (not treated: *n* = 13 for High and 8 for Low) and calves with a previous disease record in the group (treated: *n* = 9 for High and 11 for Low). (**B**) Frequency of pain-related behaviours (back-transformed means and 95% Cis (confidence intervals).) displayed by calves with no previous disease record (not treated: *n* = 13 for High and 8 for Low) and calves with a previous disease record (treated) during live observations (10 min) 3.5 h and 6 h after disbudding. Each symbol indicates †: *p* < 0.1, *: *p* < 0.05, and **: *p* < 0.001.

There was no difference between maternal treatments in the total number of head movements and kicking behaviours in response to LA administration (H: 3.9 ± 0.5, L: 3.8 ± 0.5 times, *F_1,39_* ≤ 0.1, *p* = 0.977) and disbudding (H: 6.2 ± 1.3, L: 8.3 ± 1.3 times, *F_1,35_* = 3.3, *p* = 0.076). There was a significant interaction between maternal treatment and previous disease treatment record in the behavioural responses to disbudding (*F_1,34_* = 22.5, *p* < 0.001). L calves with no previous disease treatment record in the group pen (non-treated L: *n* = 8) displayed significantly higher behavioural responses to disbudding compared to H calves with no previous disease treatment record (non-treated H: *n* = 13; *p* < 0.001) and L calves with a previous disease record in the group pen (treated L: *n* = 11, *p* < 0.001, Figure 3A). Behavioural reactions to disbudding were not significantly different between treated L and treated H (*n* = 9) calves, but treated H calves tended to show higher behavioural reactions compared to treated L calves (*p* = 0.077) and non-treated H calves (*p* = 0.060; Figure 3A). 

There were no significant differences between treatment groups or disease incidence category on the frequency of pain-related behaviours (head shaking, ear flicking and head rubbing) at 3.5 h post-disbudding (treatment: *F_1,37_* = 2.4, *p* = 0.132; disease incidence category: *F_1,37_* = 1.5, *p* = 0.223). However, a significant interaction was found between maternal treatment and disease record (*F_1,37_* = 5.8, *p* = 0.016). Non-treated L calves displayed significantly more frequent pain-related behaviours compared to non-treated H calves (*p* = 0.036), but the treatment effect was not observed in treated calves (Figure 3B). Previous experience of disease tended to reduce the expression of the pain-related behaviour in L calves (*p* = 0.051), but this was not observed in H calves (Figure 3B). At 6 h post-disbudding, L calves displayed almost twice the frequency of pain-related behaviour as H calves, but this was not statistically significant (*F_1,33_* = 3.4, *p* = 0.076). Regardless of the treatment groups, non-treated calves displayed significantly more frequent pain-related behaviours than treated calves at 6 h post-disbudding (non-treated: 20.9 [7.7–56.6]; treated: 7.8 [2.5–23.8], *F_1,36_* = 4.9, *p* = 0.033). 

**Figure 4:**
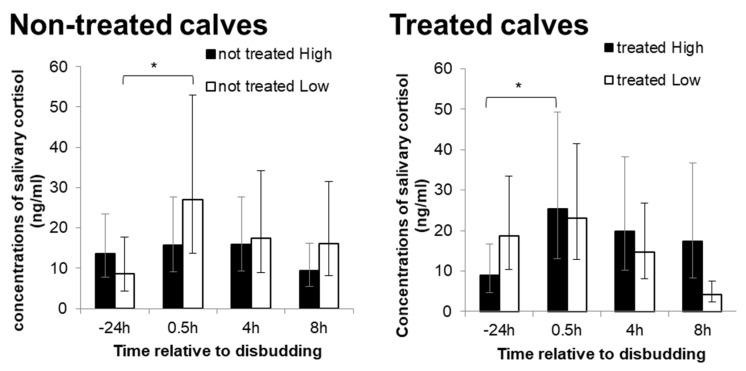
Change in the concentrations of salivary cortisol from baseline (24 h pre-disbudding) to 0.5 h, 4 h and 8 h post-disbudding (back-transformed means and corresponding 95% Cis (confidence intervals).) for calves with no previous disease record in the group pen (left) and calves with a previous disease record in the group pen (right). * indicate significant differences between baseline and 0.5 h post-disbudding within treatments (*p* < 0.05).

There was no maternal treatment effect on the salivary cortisol level at any sampling point (*F_3,140_* = 1.5, *p* = 0.216). However, a significant effect of timing was observed (*F_3,146_* = 3.2, *p* < 0.001), where the concentration of salivary cortisol significantly increased from the baseline (−24 h pre-disbudding) to 0.5 h post-disbudding (*p* = 0.008), and gradually decreased at 4 h post-disbudding. Moreover, there was a significant interaction between treatment and disease incidence in the change in salivary cortisol level (*F_3,135_* = 5.0, *p* = 0.002). Salivary cortisol levels for non-treated L calves were significantly elevated from the baseline at 0.5 h post-disbudding (*p* = 0.013), but no significant difference was observed in non-treated H calves (Figure 4). In contrast, treated H calves showed a significant increase in the salivary cortisol level from the baseline at 0.5 h post-disbudding (*p* = 0.014), whilst no significant difference was observed in treated L calves (Figure 4). At 8 h post-disbudding, the salivary cortisol levels for treated L calves were significantly decreased from 0.5 h post-disbudding (*p* < 0.001), which were significantly lower than treated H calves (*p* = 0.002).

## 4. Discussion

Calves in the current study were born to cows that experienced either high or low stocking density during the dry period [32]. We hypothesised that stressful maternal experiences during late pregnancy due to high stocking density (i.e., increased agonistic interactions at the feed face and shortened feeding time during peak feeding periods) would negatively affect foetal development. Significant effects of maternal treatment were observed in calf social behaviour and responses to weaning. Calves born to high stocking group cows made more frequent social contact with pen mates in the group pen, had lower proportions of time spent lying around weaning and increased time spent closer to the feeders. Moreover, significant interactions were found between maternal treatment and previous disease treatment record in the behavioural and physiological responses to painful procedure and pain-related behaviour. Calves’ behavioural and physiological responses to disbudding were less distinct in the high stocking group compared to the opposite group, but this effect was observed only in healthy calves. The effect of previous disease treatment record was exerted differently between the groups. In the low stocking density group, healthy calves displayed higher behavioural and physiological responses compared to calves with previous disease treatment record, but the opposite or no effect was observed in the high stocking density group. The current study, however, found no detectable impact of maternal high stocking density during late pregnancy on calf body weight, pre-weaning growth, activity levels in the first week of life, and most of the behavioural outcomes during the first three weeks of life. 

Disbudding or dehorning is commonly practiced on cattle farms, and is known to be painful [41]. Maternal high stocking density suppressed behavioural reactions of healthy calves to pain immediately and at 3.5 h after disbudding procedure. Maternal high stocking density treatment also diminished the level of increase in salivary cortisol before and after disbudding in healthy calves, although the opposite effect was observed in calves that had been treated for disease. This may suggest that maternal high stocking density reduced pain perception in healthy calves. These results contradict previous studies in other animals, which have shown that maternal stress during pregnancy increased offspring’s sensitivity to pain [42,43,44]. The different outcomes could be due to differences in the level of maternal stress, but it is unclear from the current study why the behavioural and physiological reactions to disbudding were reduced in calves from the high stocking density group. 

Compared to healthy L calves, behavioural and physiological responses to disbudding were reduced in L calves that had been treated for disease as well as healthy H calves. Healthy calves from both groups also displayed pain-related behaviour more frequently than treated calves at 6 h post-disbudding. Calves in a state of ill health have been shown to reduce exploratory and social behaviours [45,46]. This is considered an organised and adaptive behavioural strategy to conserve energy for tackling disease by increasing resting time [46,47]. Although treated calves in the current study were not necessarily ill at the time of observation, their previous health status may have affected their sensitivity to pain or the expression of pain-related behaviour. It also appears that prenatal exposure to maternal high stocking density may have had a similar impact on reactions of healthy calves to pain or sensitivity to pain as health state. The current study also indicated that ill health negatively affected calf growth and activity levels in the first week of life, which is in agreement with previous studies [48,49,50]. The current study suggests that calf health state had a greater impact on calf growth and activity levels than maternal treatment. Moreover, maternal treatment affected the reaction to disbudding differently, depending on calf health status. 

Weaning is often stressful in farm animals, as it usually occurs at an earlier age compared to natural conditions [51]. When the weaning process started, calves from both treatment groups visited milk and starter feeders more often compared to the day before weaning. This probably indicated that calves were hungry [52]. Before weaning, the lying proportion was not different between the maternal treatment groups, but only H calves decreased their lying proportion after the gradual weaning process had started. Calves are shown to become more active when feed is restricted [52] or during weaning [53], which may also be an indicator of hunger [52]. The reduced lying proportion during weaning may indicate a greater restlessness of H calves due to weaning. Calves were observed more often in the straw yard (i.e., where all the feeders were located) during the gradual weaning process, potentially suggesting that calves wanted to stay closer to the feeders during weaning. When the weaning process was completed, H calves were observed in the straw yard more often than L calves. These results suggest that maternal high stocking density treatment may have increased offspring behavioural reactions to weaning. In pigs, prenatal stress has been shown to increase the reactivity of piglets to weaning stress [54]. Our findings suggest potential associations between maternal treatment and increased behavioural reactions of offspring to weaning stress. 

Most of the behaviours observed at the introduction to the group pen and during the first two weeks in the group pen were not affected by maternal treatment. Additionally, any significant treatment differences found in group-pen behaviours were not consistent, making it difficult to interpret the results. For example, at the introduction to the group pen, calves born to cows in the high stocking density group tended to take longer to start exploring the group pen and tended to receive fewer social contacts from their companions. This may indicate fear response to a novel environment and novel companions. Calves born to cows in the low stocking density group spent less time walking and running as the group size increased, which may also indicate fear responses of these calves to an increased number of companions as lower locomotor activity is associated with fearfulness in dairy cows [55,56]. However, there were no such treatment effects on any of the other behavioural parameters, including latencies and frequencies of locomotor and social behaviours during the first 30 min in the group pen, for calves from either of the groups.

On the day following introduction to the group pen, daily lying proportions increased only for calves from the low stocking density group, which may indicate that these calves rested more on the following day. Increased resting time could be interpreted as indicative of calves from the low stocking density group acclimatising to the group environment sooner than calves from the high stocking density group. In combination with fear-associated behaviours observed at the introduction to the group pen, calves in the high stocking density group appear to take longer to adapt to a new environment with novel companions. However, there was no consistent treatment effect on other behavioural parameters, meaning that we did not find strong evidence that can support this statement.

In the first two weeks in the group pen, calves born to cows in the high stocking density group made social contact more frequently compared to the calves in the opposite group. This may indicate that calves from the high stocking density group were more social or more motivated to socialise [57,58]. Early social experience is associated with improved cognitive abilities [59] and social competency [60], which is essential for the development of social behaviour [61]. It appears that maternal high stocking treatment enhanced offspring’s social motivation. However, another parameter that is often used to assess sociability of calves (i.e., the proximity to other calves) was not affected by maternal treatment. 

To our knowledge, this is the first study in cattle that has investigated the effect of maternal social stress during late pregnancy on pre-weaned calf performance. Excessive secretion of maternal cortisol due to higher levels of stress during pregnancy is usually associated with altered behaviour of offspring, such as an increased stress reactivity and cognitive and behavioural problems [2,4]. However, we found no evidence that a high stocking density during the dry period caused a physiological stress response in dry cows [32]. The lack of significant differences in physiological stress parameters in cows may be due to a treatment setting that was not severe enough to induce physiological stress responses in cows (discussed in [32]). Indeed, behavioural alterations observed in cows were concentrated on the feeding time (once per day), which may not have imposed chronic stress to dry cows. Therefore, the circulating cortisol levels of cows in the high stocking density group may have only a minor impact on the programming of the foetal brain. This could explain the lack of measurable significant effects of maternal treatment on most of the calf outcomes measured in this study. It is still interesting to note that offspring changes occurred in the apparent absence of major change to maternal cortisol output (though the possibility that such changes did exist but were not detected by the measurements taken cannot be excluded). In the absence of data supporting alternative physiological explanations it is only possible to speculate about alternative mechanisms, such as alteration of placental function and subsequent changes in the level of maternal cortisol exposure to the foetus.

Moreover, there was a large amount of individual animal variation in maternal faecal glucocorticoid levels [32] and behavioural outcomes of the calves. Therefore, it is possible that some individual cows in the low stocking density group had higher stress levels than individual cows in the high stocking density group, and their offspring were affected accordingly. Nevertheless, the current study indicated that there were some detectable effects of maternal high stocking density treatment on calf social behaviour and reactivity to pain and weaning. These behavioural outcomes may not necessarily be mediated by maternal cortisol levels and may not necessarily be considered negative or harmful, and the possibility that these behavioural changes occurred by chance cannot be excluded. Investigations of individual cow–calf relationships may be able to find associations between prenatal experience and calf outcomes, which may have been masked by the wider group comparisons.

## 5. Conclusions

To our knowledge, this study is the first to demonstrate associations between maternal stocking density during late pregnancy and offspring behaviour in dairy cattle. The current study indicated that maternal high stocking density treatment increased calves’ behavioural reactions to weaning but reduced reactivity to pain. Mechanisms of such effects are still unclear from the current study, and further studies will be required to elucidate these. 

## Figures and Tables

**Table 1:** Definitions of locations and postures, details of behaviours and event/states used for the observation of calf reactions to a group environment (1), calf behaviour in the first two weeks in the group pen (2), and calf reactions to weaning (3).

Location	Definition	Observation
Milk feeder	At least one front foot of the calf is inside the feeder stall.	1, 2, 3
Starter feeder (group pen)	The calf’s head is within a half body length from the line between the starter feeder and the straw-bedded area.	2
Starter feeder (weaning)	The calf’s head is fully across the line between the straw-bedded area and the starter trough, and remained there for more than five seconds. The starter feeder visit is finished when the calf’s head is away from the starter trough for more than ten seconds.	3
Straw yard	At least one foot of the calf is in the straw-bedded area.	1, 2, 3
Igloo	Four legs of the calf cross the front line of the igloo.	1, 2, 3
Water dispenser	The muzzle of the calf touches or is within 5 cm from the water dispenser.	3
**Posture**		
Standing inactive	The calf is in an upright position with no leg movement.	1
Standing	The calf is in an upright position including walking and running.	2, 3
Lying	The calf lies down with sternal recumbency or lies on its flank.	1, 2, 3
Walking	The calf makes a forward movement with more than two steps. Two or three hoofs are touching the ground at any time.	1
Running	The calf makes a rapid forward movement, including instances of jumping, bucking, galloping and trotting [35,36].	1
**Behaviour**		
Explore	The muzzle of the calf is in contact with or within approximately 5 cm from the wall, floor, chain, straw feeder, water dispenser, starter or any other objects in the pen.	1
Social contact initiated	The muzzle of the calf is in contact with or within approximately 5 cm from the head or body of another calf.	1
Social contact received	The muzzle of another calf (calves) is (are) in contact or within 5 cm from any parts of the focal calf (lasting 3 s or more).	1
No contact	The muzzle of the calf is not in contact with any objects in the pen, any parts of another calf’s body or its own body.	1
**Touch**		
Social touch actor	The muzzle of the focal calf is in contact with any parts of another calf. Head and neck oriented towards recipient.	2
Social touch recipient	The muzzle of other calf (calves) is/are in contact with any body parts of the focal calf.	2
**Proximity**		
Close	Distance to neighbouring calf (calves) is within one body width.	2
Not close	Distance to neighbouring calf (calves) is more than one body width.	2
**Event/state**		
Human presence	There are human(s) inside or outside of the front face of the pen.	1, 2, 3 *

* Inside only.

**Table 2:** Body weights (kg) at birth, at introduction to the group and weaning, average daily gains (ADG: g/day) in the hutch, the group and the pre-weaning period, IgG (Immunoglobulin G) levels, and daily lying proportion in the hutch (LP hutch) and in the group pen (LP group). The figures indicate means ± SEM.

Measurement		High Stocking	Low Stocking	Test Statistics	*p*-Value
Body weight (kg)	birth	45.4 ± 1.0	46.5 ± 1.1	*W* = 0.6	0.439
	day 7	45.1 ± 0.9	46.1 ± 1.0	*W* = 0.5	0.492
	wean	69.0 ± 1.5	69.8 ± 1.7	*W* = 0.1	0.759
ADG (g/day)	hutch	−25.6 ± 57.1	−5.3 ± 62.6	*W* = 0.1	0.812
	group	578.7 ± 27.1	587.1 ± 32.0	*W* < 0.1	0.844
	pre-weaning	493.2 ± 25.8	489.0 ± 30.3	*W* < 0.1	0.915
IgG levels (mg/mL) on day 7		7.9 ± 0.8	8.1 ± 0.9	*W* < 0.1	0.911
LP hutch	day 2	0.88 ± 0.02	0.90 ± 0.02	Treatment: *F_1,34_* = 1.0	0.338
	day 3	0.86 ± 0.02	0.87 ± 0.02	Day: *F_4,125_*_=_ 9.4	<0.001
	day 4	0.85 ± 0.02	0.85 ± 0.02		
	day 5	0.84 ± 0.02	0.84 ± 0.02		
	day 6	0.84 ± 0.02	0.86 ± 0.02		
LP group	group day 1 *	0.82 ± 0.02	0.80 ± 0.02	Treatment × Day: *F_1,33_* = 6.0	0.019
	group day 2 *	0.81 ± 0.02	0.83 ± 0.02		

* Group d1: 0–24 h in the group pen; group d2: 24–48 h in the group pen.

## Supplementary Materials

The following are available online at https://www.mdpi.com/2076-2615/10/6/922/s1, Table S1: Median latencies (IQR) calculated from raw data for each of the behaviours and hazard ratios [95% CIs] of L calves in performing each of the behaviours during the first 30 min in the group pen, Table S2: Time spent on each of the behaviours, the location (duration) and the frequency of each of the behaviours observed in the first 30 min in the group pen.
