# Supplementary material for: Impact of Maternal High Stocking Density during the Dry Period on Dairy Calf Health, Behaviour, and Welfare
Source: Animals (Basel). 2020 May 26;10(6):922. doi: 10.3390/ani10060922 (PMC7341238; doi:10.3390/ani10060922)
Supplement: Supplementary file 1 [file animals-10-00922-s001.pdf]

animals

MDPI

Supplementary Files: Impact of Maternal High Stocking Density during the Dry Period on Dairy Calf Health, Behaviour, and Welfare

Mayumi Fujiwara, Marie J Haskell, Alastair I Macrae and Kenneth MD Rutherford

Table S1. Median latencies (IQR) calculated from raw data for each of the behaviours and hazard ratios [95% CIs] of L calves in performing each of the behaviours during the first 30 minutes in the group pen.

Behaviour	Median (Seconds) (IQR)		Hazard Ratio [95% Cis]	P-Value
	High Stocking	Low Stocking	Reference =H Group	
Walk	6.6 (3.0–13.2)	8.4 (3.6–23.4)	0.56 [0.23−1.33]	0.186
Run	46.8 (24.6–115.8)	42.0 (33.6–160.2)	0.86 [0.36−2.04]	0.728
Explore	24.0 (15.0–35.4)	16.2 (7.8–97.8)	2.13 [0.97−4.66]	0.061
Initiate social contact	16.2 (7.8–97.8)	21.0 (6.0–69.6)	0.69 [0.30−1.58]	0.381
Receive social contact	68.4 (15.6–195.6)	87.6 (34.8–217.8)	1.24 [0.56−2.72]	0.598
**N calves performed (%)**				
Lie down *	19/20 (95.0)	13/15 (86.7)	1.63 [0.55−4.89]	0.380
Enter the igloo shed *	9/20 (45.0)	8/15 (53.3)	1.17 [0.49−2.77]	0.728

* Censoring occurred for the latencies to lie down and enter the igloo shed, hence medians could not be calculated. Number and percentage of calves performing the behaviours are presented.

Table S2. Time spent on each of the behaviours, the location (duration) and the frequency of each of the behaviours observed in the first 30 minutes in the group pen. Figures indicate predicted means ± SEM or back-transformed means [95%CIs] for the data analysed following a log-transformation.

Behaviour	High Stocking	Low Stocking	W Statistic	p-Value
**Duration (minutes)**				
Standing inactive	7.7 ± 1.2	8.0 ± 1.2	0.1	0.722
Walking	5.6 ± 0.4	5.9 ± 0.5	0.2	0.686
Running	1.6 ± 0.4	1.4 ± 0.4	0.1	0.729
Exploring	7.5 ± 1.2	8.9 ± 1.2	1.7	0.257
Social interaction	1.1 [0.7–2.0]	0.8 [0.5–1.5]	1.9	0.179
Straw yard	17.9 ± 2.3	18.7 ± 2.6	0.1	0.725
Igloo shed	10.3 ± 2.6	9.8 ± 2.8	0.1	0.833
**Frequency (count/30 min)**				
Walking	44.9 ± 7.4	48.1 ± 8.8	0.2	0.638
Running	20.9 ± 3.8	21.4 ± 4.0	<0.1	0.923
Exploring	47.0 ± 8.2	56.9 ± 9.8	1.8	0.194
Exploring (straw yard)	25.1 ± 6.2	35.6 ± 7.1	2.6	0.117
Exploring (igloo shed)	19.5 ± 4.8	17.8 ± 5.1	0.1	0.822
Social contact initiated	12.9 ± 4.0	10.9 ± 4.3	0.3	0.570
Social contact received	12.8 ± 1.7	17.1 ± 2.0	4.2	0.051
